# Validating a Four-Factor Model of Psychopathic Personality from the Triarchic Psychopathy Measure (TriPM) Across Community and Incarcerated Samples †

**DOI:** 10.3390/bs15111503

**Published:** 2025-11-05

**Authors:** Sandeep Roy, Mariia Mezhenska, Craig S. Neumann, Nicola S. Gray, Robert J. Snowden

**Affiliations:** 1 Department of Psychology, San Antonio State Hospital, San Antonio, TX 78223, USA; 2 Department of Psychology, University of North Texas, Denton, TX 76205, USA; mariiamezhenska@my.unt.edu; 3 Department of Psychology, Swansea University, Swansea SA2 8PP, UK; nicola.s.gray@swansea.ac.uk; 4 Department of Psychology, Swansea Bay University Health Board, Bridgend CF31 4LN, UK; 5 School of Psychology, Cardiff University, Cardiff CF10 3NB, UK; snowden@cardiff.ac.uk

**Keywords:** psychopathy, PCL-R, SRP-SF, TriPM, measurement invariance, SEM

## Abstract

The Triarchic Psychopathy Measure (TriPM) is based on a three-dimensional conceptual model, though structural analyses of the TriPM items indicate that they do not reflect this conceptual model. In contrast, studies have shown that multiple factors are required to account for all the TriPM items in community and incarcerated samples. More problematic is that some of these factors are outside of the nomological network of psychopathy. In contrast, there are empirically robust findings supporting the four-factor model of psychopathy, irrespective of sample type, assessment method, or item set. For the current study, a structural equation modeling approach was utilized with incarcerated and community samples to demonstrate that theoretically relevant candidate items from the TriPM could be employed to represent the four-factor model of psychopathy (i.e., four-factor proxy measure—4FPM). Multiple group confirmatory factor analysis of the 4FPM items provided evidence of strong (scalar) invariance across community and incarcerated samples. Finally, associations with external correlates and other psychopathy scales highlighted that the 4FPM can be utilized to represent the four-factor model of psychopathy.

## 1. Introduction

Psychopathic personality is a construct of considerable relevance to mental health and criminal justice systems, given its fundamental associations with violent and dissocial behavior (Gray et al., 2021; Hare et al., 2018; Neumann & Hare, 2008; Olver et al., 2020). The Psychopathy Checklist-Revised (PCL-R; Hare, 2003) is widely considered the international standard for the clinical and forensic assessment of psychopathic personality (De Brito et al., 2021). The PCL-R and its derivatives, such as the Self-Report Psychopathy Scale (SRP; Paulhus et al., 2017), have been employed to statistically represent the multifarious syndrome of psychopathy as a dimensional superordinate construct underpinned by four correlated first-order factors (Hare & Neumann, 2008; Neumann et al., 2007; Neumann & Hare, 2008). The strength of the four-factor model is that it holds up to rigorous latent variable modeling across different item sets, assessment tools, and sample types (Neumann et al., 2015).

The four PCL-based factors are the following: *Interpersonal* (grandiose, deceptive, and manipulative interpersonal style), *Affective* (diminished empathy, remorse, and affiliative emotions), *Lifestyle* (impulsive, parasitic, disloyal, and erratic), and *Antisocial* (low frustration tolerance, inherent and longstanding disregard for social and conventional norms, not necessarily criminal; see Hare & Neumann, 2010). The unidimensional first-order factors have been shown to evidence differential links with various external correlates, such as traumatic experiences and adverse events (*rs* = −0.11 to 0.27, Graham et al., 2012), emotion regulation (*β* = 0.32, Garofalo et al., 2020; *rs* = −0.43 to 0.44, Love & Holder, 2014), and alcohol and substance use (*rs* = 0.18 to 0.27, Neumann & Hare, 2008; *rs* = 0.14 to 0.44, Potik et al., 2019), allowing for a more dynamic parsing of the nomological network of psychopathy. However, the superordinate factor, which is optimal for identifying individuals using total cut-scores (Neumann et al., 2007), has also demonstrated utility by linking this broad (syndromal) factor to increased alcohol use, elevated violence, diminished scores on intellectual testing, and decreased paralimbic system gray matter volume (Baskin-Sommers et al., 2016; Kim et al., 2024; Neumann & Hare, 2008). Taken together, the superordinate factor and the unidimensional first-order factors of the PCL-based scales provide varying levels of analysis to help delineate the correlates of psychopathic personality.

Another perspective on psychopathic personality is the Triarchic Model of Psychopathy (Patrick & Drislane, 2015). The Triarchic Model is proposed to tap *three* neuro-biobehavioral trait domains: Boldness, Meanness, and Disinhibition (Sleep et al., 2019). Disinhibition reflects a wide diversity of impulse control difficulties (Patrick & Drislane, 2015), including overt antisociality (Roy et al., 2021a). Meanness taps multiple domains, including deficient empathy, lack of close attachments, and disdain for and exploitation of others (Patrick & Drislane, 2015), whereas Boldness is thought to reflect a large diversity of trait domains involving confidence, social assertiveness, fearlessness, emotional resiliency, and adventuresomeness (Patrick & Drislane, 2015). In contrast to the broad (superordinate) syndrome indexed by the PCL scales, Patrick et al. (2012, p. 256) suggest that “the three components of the Triarchic Model are not considered elements or indicators of a unitary higher order psychopathy construct.” Instead, the Triarchic constructs are considered “building blocks for alternative conceptions of variants of psychopathy described by historic and contemporary writers” (Patrick et al., 2012, p. 256). Proponents of this model also assert that the Triarchic constructs transcend assessment instruments or psychopathy theory, as evidenced by efforts to create Triarchic scales from a variety of psychopathy and normal-range personality instruments (Collison et al., 2021). This perspective is reflected in the creation of the Triarchic Psychopathy Measure (TriPM; Patrick, 2010), which was built by taking items from the Boldness Inventory (Patrick et al., 2019) and the Externalizing Spectrum Inventory (ESI; Krueger et al., 2007) to create three scales indexing the Triarchic constructs. Despite some evidence in support of the TriPM scales, Sleep et al. (2019) questioned the relevance of Boldness to the construct of psychopathy, as well as the poor discriminant validity between the Meanness and Disinhibition scales, which they attributed to issues regarding the internal structure of the TriPM in their meta-analytic review of the nomological network of the Triarchic Model of Psychopathy. Also, notwithstanding the Triarchic Model not lending itself to a broad syndrome, researchers have nonetheless employed TriPM total scores to identify individuals with broadly elevated psychopathic propensities (e.g., Drislane et al., 2014).

### 1.1. Latent Variable Analyses of Psychopathy Measures

Sophisticated structural analyses of the items from various psychological measures are essential to evaluating whether the items empirically conform to the theoretical structure of the construct they are intended to index (Roy et al., 2021b). Such analyses can inform whether the use of manifest (i.e., observed) scores on a measure is congruent with the latent (i.e., unobserved) construct being assessed. In the context of psychopathic personality, latent variable modeling provides quantitative information on how well items discriminate individuals who vary in psychopathic propensities, and which items are crucial for modeling the hypothetical domains they are designed to tap (Hare & Neumann, 2008; Roy et al., 2021a). Moreover, only item-level modeling, particularly analyses focused on examining the extent to which a latent construct is being measured equivalently across groups or over time (i.e., measurement invariance; Maassen et al., 2023), can be used to provide the statistical assurance for comparisons of various groups^1^. Regarding the PCL scales, measurement invariance has been found for the four-factor PCL model across culture (Neumann et al., 2006), gender (i.e., Neumann et al., 2012; Walsh et al., 2019), development (Bellamy et al., 2024; Ngo et al., 2024), and intellectual functioning (Kim et al., 2024). Findings such as these indicate that the same underlying construct is being measured in a similar manner across diverse demographic and developmental samples. Maassen et al. (2023, p. 3) noted that “without [measurement invariance testing], researchers remain in the dark about whether observed differences between groups are due to measurement bias, which also undermines the generalizability of the findings.” In the context of psychopathy, where legal questions are often raised, it is critical to establish that such measures assess the intended hypothetical construct in a consistent manner across diverse sample demographics.

Only a small set of TriPM studies to our knowledge have examined the item-level properties of the measure and have found contrasting results. Generally speaking, the studies supporting a three-factor model of the TriPM (Latzman et al., 2019; Paiva et al., 2020; Patrick et al., 2021; Somma et al., 2019) employ a combination of bifactor modeling, exploratory structural equation modeling, and a large number of correlated residual error terms; an analytic approach that diverges considerably from the practical scoring and use of the TriPM (Roy et al., 2021b). In contrast, other studies have consistently found, and independently validated, support for six to seven factor models to account for the TriPM item set through a combination of exploratory and confirmatory factor analytic approaches in community and incarcerated samples (Collison et al., 2021; Pink et al., 2022; Roy et al., 2021a; Stanton et al., 2021; Yao et al., 2025). Although these alternative models of the TriPM improved prediction of external correlates, some of the identified factors are not anchored in existing models of psychopathy, which makes integrating these findings into the nomological network of the construct challenging. Additionally, only two of the studies examining the item-level structure of the TriPM have examined measurement invariance with divergent analytic approaches and subsequent results (Pink et al., 2022; Somma et al., 2019), highlighting the need for further exploration of the measurement invariance of the TriPM item set.

Considering the associations the TriPM evidence with other psychopathy measures, including the PCL measures (Sleep et al., 2019), as well as the diversity of content inherent in the scales, it is plausible that some items of the TriPM may be reorganized to create scales aligned with the four-factor PCL-based model of psychopathic personality. Deriving a four-factor model based on relevant TriPM items would allow investigators to examine the psychometric properties and practical utility of two distinct yet overlapping frameworks for understanding psychopathic personality using one item set (PCL-based & Triarchic). Such a scale would serve as a useful complement to the TriPM, given the ongoing debates over the measurement properties of its original scales, as well as facilitate continued research of the four-factor model in datasets that employ the TriPM.

### 1.2. Current Study

Using both community and incarcerated samples, in conjunction with a latent variable modeling approach, the current study was carried out to explore whether theoretically relevant items from the TriPM can be utilized to construct four unidimensional factors reflecting the PCL-based four-factor model of psychopathy (i.e., the 4FPM) and whether the model employing the item set was invariant across community and incarcerated samples. For comparative purposes, we also modeled the Self-Report Psychopathy-Short Form (SRP-SF; Paulhus et al., 2017), in conjunction with the 4FPM, though note that the SRP-SF was only available for the community sample. In addition, we had access to the PCL-R scales, but only for the incarcerated sample, which were correlated with the 4FPM domains. To be clear, the goal of the study was not to develop a scale to replace the original TriPM scales or examine their psychometric properties, as such work has been performed previously (e.g., Collison et al., 2021; Roy et al., 2021a; Somma et al., 2019; Stanton et al., 2021). Rather, it was to assess if some of the TriPM items can be configured into scales reflecting the four-factor model of psychopathic personality indexed by the PCL scales (Neumann et al., 2015).

Construct validity of the 4FPM was examined via its profile similarity (i.e., pattern of latent and manifest correlations with external correlates) with the SRP-SF and external correlates in the community sample and through bivariate correlations with PCL-R scale scores in the correctional sample. The following hypotheses were proposed:

(1) The 4FPM would evidence acceptable model fit in the community and incarcerated samples.
(2) The 4FPM and SRP-SF facets would display highly congruent (i.e., similar direction and magnitude) patterns of associations with external correlates and profile similarity in the community sample. Specifically, it was anticipated that all domains of the 4FPM and SRP-SF would be positively associated with alcohol use and negative affect and negatively associated with positive affect. It was also anticipated that the Lifestyle and Antisocial facets would be positively associated with trauma history, whereas the Interpersonal and Affective facets associations with trauma history would be negligibly small.
(3) The four factors of the 4FPM would display convergent validity with their analogous scales on the PCL-R in the incarcerated sample.
(4) The four latent factors of the 4FPM would load onto superordinate psychopathy factors in the community and incarcerated sample, consistent with prior PCL-R research (Neumann et al., 2007).
(5) The superordinate psychopathy factor of the 4FPM and the SRP-SF would evidence comparable latent regression associations (i.e., similar magnitude and directions) with relevant external correlates in the community sample. Specifically, we expected positive associations with alcohol use and negative affect, negative associations with positive affect, and marginal associations with trauma history, considering the divergent facet associations.

All hypotheses were formulated prior to data analysis without preregistration. No specific hypotheses regarding measurement invariance or group differences were formulated for the 4FPM, given the exploratory nature of the measure. Although not the primary focus of this project, we also expected that the four facets of the SRP-SF would display acceptable model fit, evidence strong measurement invariance across gender in the community sample, consistent with prior SRP-SF findings (Neumann et al., 2012), and load onto a superordinate psychopathy factor in the community sample. This article is a revised and expanded version of a paper entitled “Three- Seven- or Four-factor Structure of the Triarchic Psychopathy Measure?,” which was presented at the seventh biennial meeting for the Society of the Scientific Study of Psychopathy in Antwerp, Belgium, in 2017 (Neumann et al., 2017).

## 2. Method

### 2.1. Sample Description and Measures

The community and correctional samples used in the current study were previously examined in research conducted by Roy et al. (2021a, 2021b) investigating the latent structure of the Triarchic Psychopathy Measure (Patrick, 2010). Institutional approval and ethical review of research protocols and data collection methods were obtained from the University of North Texas Institutional Review Board, the Grendon Research and Advisory Committee, and the NISCHR Wales Research Ethics Committee. Informed consent was obtained from all participants in both samples examined in the study.

**Community Sample.** The community sample was composed of 1064 participants from Amazon’s Mechanical Turk Platform (MTurk; 53% male, *M_age_* = 34.12 years, *SD* = 10.49). Participants were White (77%), African American (7.4%), Hispanic (5%), Asian (6.2%), or other (4.6%) with a 4-year college degree (38%), some college (30%), 2-year college degree (10%), high school degree (9.3%), or graduate education (12.7%). The integrity of the data was screened via validity check items. Participants’ data were only included if all four validity questions were answered correctly (98% of the sample). Reliability estimates (i.e., Cronbach Alpha [*α*], mean inter-item correlations [*MIC*]) of the TriPM scales were acceptable in this sample (*α*’s = 0.87 to 0.90, *MIC*’s = 0.27 to 0.33).

**Correctional Sample.** The correctional sample was composed of 159 British men (*M_age_* = 45; 76% White, 5% Black, 19% mixed or other race/ethnicities; 80% either single or separated/divorced). A total of 40% had achieved General Certificate of Education (GCE) Ordinary Level, 7.1% achieved A-levels, 1.4% formal degrees, 8.6% some other form of certificate through prison, and 42% had no formal education. Index offenses for the sample included murder, attempted murder, rape, wounding, grievous bodily harm, robbery, and other offenses. Indices of internal consistency for the TriPM scales in the incarcerated sample generally fell within the acceptable range (*α*’s = 0.80 to 0.91, *MIC*’s = 0.17 to 0.36).

**External Correlates.** External correlates from the community sample were the following: the Positive and Negative Affective Schedule (Watson et al., 1988), the Alcohol Use Disorders Identifications test (Saunders et al., 1993), and the Trauma History Questionnaire (Green, 1996). Of note, the Self-Report Psychopathy Scale-Short Form (SRP-SF; Paulhus et al., 2017) was administered in the community sample, though it was not comprehensively examined in the prior studies utilizing this dataset. The SRP-SF is a 29-item self-report scale used to assess psychopathic traits. It has a latent structure consistent with the PCL-R four-factor model (Paulhus et al., 2017; Neumann et al., 2015). Items are rated on a 5-point Likert scale, ranging from 1 (*Disagree Strongly*) to 5 (*Agree Strongly*). Additionally, the incarcerated sample contained the Psychopathy Checklist-Revised (PCL-R; Hare, 2003), which was not previously examined in the series of studies conducted by Roy et al. (2021a, 2021b). The PCL-R contains 20 items, each rated on a three-point scale (0, 1, and 2) according to the extent to which an item description matches the individual (Hare, 2003). Total scores can vary from 0 to 40. The incarcerated sample contained no other external correlates.

### 2.2. Data Analytic Plan

A structural equation modeling (SEM) approach was employed in the current study, given its methodological rigor and capacity to provide evidence of construct validity (Strauss & Smith, 2009). First, utilizing the prior psychometric investigation of the TriPM item set (Roy et al., 2021a) as well as their expertise in modeling the PCL scales, the senior author (CSN) identified 23 items across the three TriPM scales that aligned conceptually with the personality propensities indexed by the four factors captured by the respective PCL domains. For example, five items from the three Triarchic scales, which overtly assessed manipulative, deceptive, and dominant interpersonal proclivities, were set to load onto an Interpersonal latent factor. Candidate items from the TriPM item set were selected in this manner to index the 4FPM domains. Reverse-keyed items were avoided, given that they have suboptimal psychometric properties relative to positively worded items (Crego & Widiger, 2014; Zhang et al., 2016). The 4FPM model, as well as the SRP-SF model in the community sample, was tested via confirmatory factor analysis (CFA).

To ascertain the similarity between the correlational profile of the SRP-SF scales and their corresponding 4FPM derivate scales, double-entry correlations as well as analyses of shape similarity (i.e., Pearson correlation between both correlational patterns for each psychopathy scale), elevation dissimilarity (i.e., the absolute value of the difference between the two profile’s mean scores), and scatter dissimilarity (i.e., the absolute value of the difference between the two profiles’ variances) were computed using the manifest and latent correlations between the SRP-SF and 4FPM as well as the available external correlates in the community sample in accordance to Furr’s (2010) recommendations for profile similarity analysis^2^.

To examine the superordinate nature of the psychopathy measures (4FPM, SRP-SF), each of the respective four first-order psychopathy factors was specified to load onto a second-order (superordinate) factor to determine the amount of variance it accounted for in each of the first-order factors. In this same model, we also assessed how the superordinate factor accounted for variance in a latent alcohol factor (represented by the AUDIT subscales) and the summed manifest scores from the THQ and the PANAS scales in the community sample. The purpose of these analyses was to examine whether the broad psychopathy construct related to external correlates in a similar manner across two forms of measurement (i.e., SRP-SF, 4FPM).

We evaluated measurement invariance by utilizing a strong measurement invariance approach in line with our previous research (Ngo et al., 2024; Walsh et al., 2019; Kim et al., 2024). Specifically, we tested whether there was evidence of scalar measurement invariance of the 4FPM items across four groupings: (1) the entire community sample with the incarcerated sample, (2) men and women in the community sample, (3) men in the community sample with the incarcerated men, and (4) the women in the community sample with the incarcerated men. To assess for scalar invariance, the items of the 4FPM were set to load on their hypothesized factors in a multiple group confirmatory factor analysis (MGCFA), with factor loadings and threshold parameters constrained to be equal in each of the groupings. For the SRP-SF, an MGCFA was conducted between the men and women in the community sample. The invariance models were statistically compared to configural models in which the same general model was tested but without constraining item loadings or thresholds. To compare these models, we did not rely on the traditional chi-square difference test since large N’s produce significant χ^2^ values, even when the discrepancies between the two models are trivial (West et al., 2012). West and colleagues suggest using guidelines propounded by Cheung and Rensvold (2002) to assess statistical differences in model fit. If the incremental change in the comparative fit index (ΔCFI) between one model and a (nested) more-constrained model is ≤0.01, then the two models do differ in statistical fit.

Assuming evidence for strong (scalar) invariance, we examined mean differences between the samples on the original TriPM scale and the new 4FPM scales to examine if the different arrangements of the TriPM items are associated with meaningful score differences between genders and sample settings. We also examined whether the total scores of the TriPM and the 4FPM could distinguish the community and incarcerated samples. Finally, we compared 4FPM scores for individuals elevated on PCL-R total scale to those below the recommended European cut-score (<25) in the incarcerated sample (Hare et al., 2018).

To assess model fit, a two-index strategy was adopted (Hu & Bentler, 1999). For the index of relative fit (i.e., how well the structured model fit relative to an unstructured model), we used the comparative fit index (CFI). For reporting absolute model fit (i.e., how well the structured model reproduces the observed data), we used the Root Mean Square Error of Approximation (RMSEA) index and the Standardized Root Mean Residual (SRMR). We relied on the traditional Relative Index (i.e., CFI) ≥ 0.90 and Absolute Index (i.e., RMSEA, SRMR) ≤ 0.08 as indicative of acceptable model fit to avoid falsely rejecting viable latent variable models (Marsh et al., 2004). All analyses were conducted in Mplus with the robust weighted least squares (mean and variance adjusted) procedure (WLSMV) for parameter estimation and assessing model fit, given the ordinal nature of the items (Muthén & Muthén, 2017). Note that Supplementary Tables S1 and S2 provide latent and manifest correlations among the study variables for the two samples.

## 3. Results

### 3.1. CFA Results: 4FPM

Model fit for the 4FPM was generally acceptable for both the community sample (CFI = 0.94, RMSEA = 0.05, SRMR = 0.06) and the incarcerated sample (CFI = 0.94, RMSEA = 0.07, SRMR = 0.09). All factor loadings (range = 0.60–0.94) and correlations among the factors (range = 0.53–0.82) were significant (*ps* < 0.001) for both the correctional sample and the community sample (loadings range = 0.30–0.88; correlations range = 0.70–0.86, *ps* < 0.001). The standard errors for the 4FPM parameters in the community (*M_se_* = 0.02, range = 0.02–0.03) and incarcerated (*M_se_* = 0.05, range = 0.02–0.07) samples were small, indicating the robustness of the parameter estimates.

### 3.2. Profile Similarity Analyses

Supplementary Table S1 displays the latent and manifest correlations between the 4FPM scale, the SRP-SF, and the external correlates in the community sample. Supplementary Table S2 displays the latent correlations amongst the 4FPM scales and their manifest correlations between the prorated PCL-R facet scales and PCL-R total score in the incarcerated sample. Table 1 contains the results of the profile similarity analyses conducted in the community sample, which demonstrated robust similarity across all indices (e.g., *rICC* range = 0.91–0.95) between the correlational patterns of the SRP-SF scales and the respective 4FPM scales.

### 3.3. SEM Results: Superordinate Model

The superordinate model had an acceptable fit for the SRP-SF (CFI = 0.9l, RMSEA = 0.07, SRMR = 0.06) and the 4FPM (CFI = 0.92, RMSEA = 0.06, SRMR = 0.05) in the community sample. The superordinate model fit similarly for the incarcerated sample (CFI = 0.94, RMSEA = 0.08, SRMR = 0.09) and accounted for the majority of variance in each of the four first-order factors. Table 2 displays the standardized SEM parameters (β) and variance accounted for (*R*^2^) by the superordinate factor in the four psychopathy scale factors, as well as the external correlates in the community sample. For both models, the psychopathy (syndromal) superordinate factor accounted for most of the variance in each of the first-order factors. Regarding the external correlates, the SEM parameters and variance accounted for by the psychopathy superordinate factor for both 4FPM and SRP-SF models were nearly identical. The superordinate psychopathy factor was moderately associated with increased alcohol use and negative affectivity, minimally correlated with less positive affect, and negligibly linked to trauma history, whether measured by the SRP-SF or the 4FPM scales.

### 3.4. Exploratory MGCFA Results

**Figure 1:**
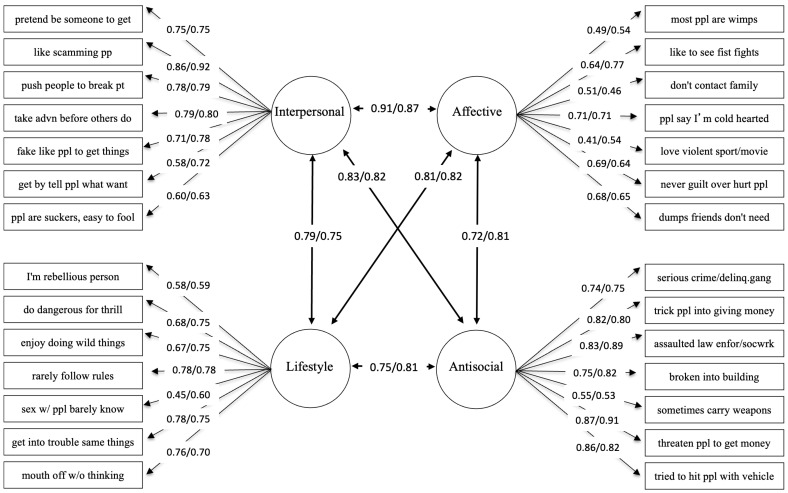
Multiple group confirmatory factor analysis results: standardized parameters (men/women) for the four-factor SRP-SF model.

With respect to invariance testing for the 4FPM items, as shown in Table 3, three of the four MGCFAs (i.e., community men and community women, overall community and incarcerated sample, community men and incarcerated men) evidenced adequate fit and strong (scalar) invariance. There was also evidence for scalar invariance for the SRP-SF items (community men and women). Evidence of strong (scalar) invariance between the female community sample and the incarcerated men was not supported. See Table 4 and Figure 1 for factor loadings across gender and sample for the 4FPM and SRP-SF, respectively. All item-to-factor loadings were statistically significant (*ps* < 0.001). The standard errors for the 4FPM parameters in the measurement invariance analyses (community and incarcerated samples: *M_se_* = 0.05, range = 0.02–0.07; community men and women: *M_se_* = 0.02, range = 0.02–0.03; community men and incarcerated men: *M_se_* = 0.04, range = 0.02–0.07; and community female and incarcerated men: *M_se_* = 0.04, range = 0.02–0.07) were small, illustrating the precision of the parameter estimates.

### 3.5. Exploratory Sample Mean Comparisons

Table 5 presents the differences in mean scores for the original TriPM and the 4FPM scales between the community and incarcerated samples. For the original TriPM scales, the incarcerated sample had lower Boldness scores and Meanness scores than the community sample, but higher Disinhibition scores. For the new 4FPM scales, the incarcerated sample had higher Affective, Lifestyle, and Antisocial scores than the overall community sample. There was no difference on the 4FPM Interpersonal scale between the incarcerated and community samples.

Regarding gender differences, men (regardless of sample setting) evidenced higher scores on the TriPM and 4FPM relative to community women, though caution is warranted in interpreting the mean score differences between the community women and the incarcerated men sample, as strong invariance was not supported in this comparison.

**Figure 2:**
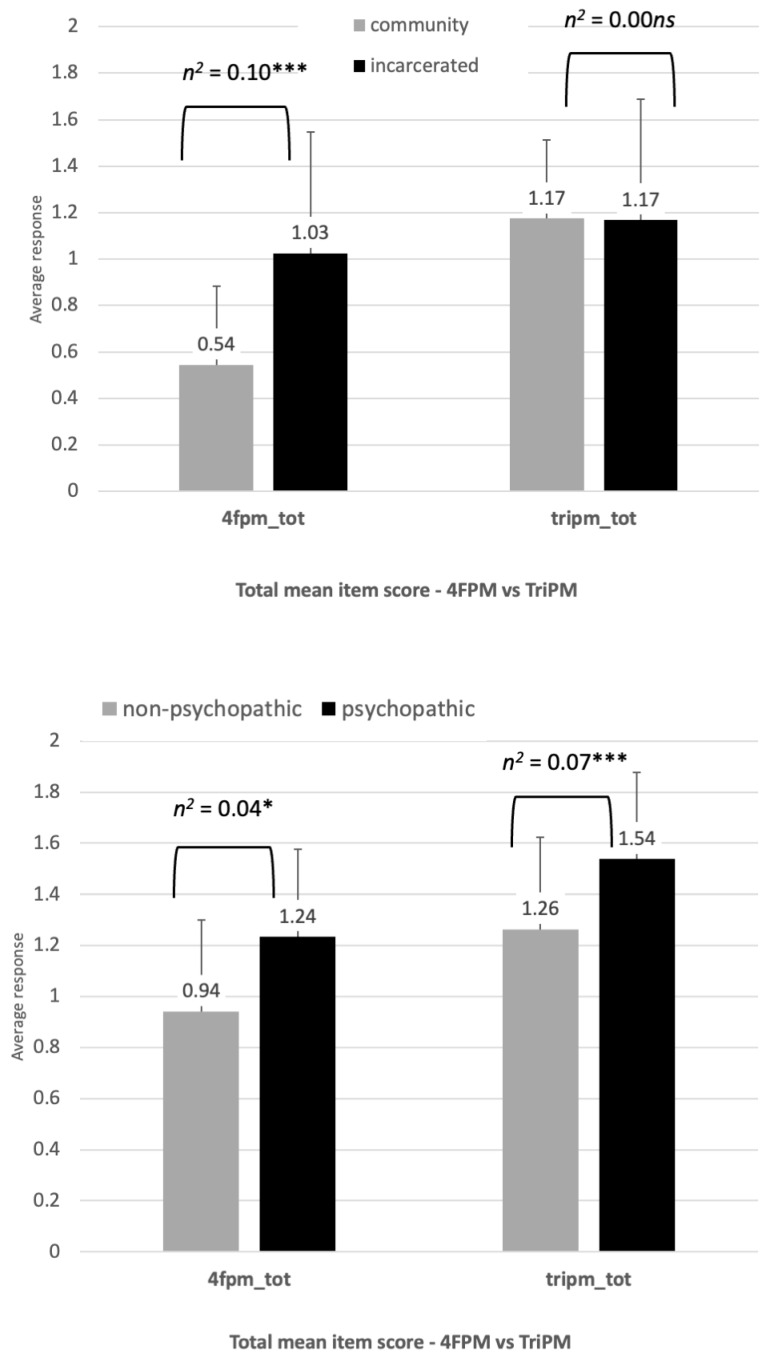
4FPM vs. TriPM total score (scaled as mean item response) comparisons between the community and incarcerated sample (**top**) and individuals with elevated PCL-R ratings (i.e., PCL-R total > 25) and those with low PCL-R ratings (**bottom**). * = *p* < 0.05; *** = *p* < 0.001.

Figure 2 displays the 4FPM total and the TriPM total score for the community and incarcerated samples (top panel), as well as those with elevated PCL-R scores from those with lower PCL-R ratings (bottom panel) for the incarcerated sample. The 4FPM total score significantly differed between the community and incarcerated samples, in contrast to the original TriPM total score, which could not distinguish the community from the incarcerated sample. Both the original TriPM total score and the 4FPM total score significantly differed between those elevated on the PCL-R versus those below the European cut-score.

## 4. Discussion

The study examined whether theoretically relevant items from the Triarchic Psychopathy Measure could serve as indicators for the domains (i.e., Interpersonal, Affective, Lifestyle, Antisocial) of the PCL-based four-factor model of psychopathy (Hare & Neumann, 2008; Neumann et al., 2015). The four-factor proxy measure (4FPM) was derived from items across all scales of the TriPM, which reflected the traits and tendencies captured by the four-factor model, in line with research showing that this model generalizes across diverse sample types, assessment methods, and item sets (Neumann et al., 2015). The 4FPM demonstrated adequate statistical fit for a correlated four-factor latent variable model across both community and incarcerated samples, and across both men and women in the community sample. The model fit statistics were similar to those of the SRP-SF. With respect to the 4FPM discrimination parameters, the majority of selected TriPM items demonstrated robust factor loadings. The items used to represent the Affective factor performed exceptionally well (community mean = 0.78; incarcerated mean = 0.83), and most of the items for the Lifestyle, Antisocial, and Interpersonal items played a prominent role in representing the domains of the four-factor model. Nonetheless, the lone item taken from the Boldness scale (i.e., “I can convince people…”) for the Interpersonal scale and one item from the Disinhibition scale (i.e., “act on immediate needs…”) for the Lifestyle scale displayed relatively diminished factor loadings in the model across genders and sample settings relative to the other selected items. One possible explanation is that neither item appears to overtly reflect dissocial tendencies or negative outcomes relative to the other 4FPM candidate items. As such, these items may evidence less utility in indexing the PCL-R model of psychopathy, which conceptualizes all dimensions and expressions of the disorder as pathological in nature (Hare et al., 2018).

Concerning construct validity, profile analyses indicated that the 4FPM facet scales evidenced comparable correlational patterns as their corresponding SRP-SF scale, signaling that these scales functioned in a similar manner in the community sample despite comprising different items. Of note, Supplementary Table S1 shows that the large correlations between the 4FPM and the SRP-SF exceeded what was documented in a meta-analysis of the Triarchic Model of Psychopathy (Sleep et al., 2019). Although this may be partially attributable to the latent variable analyses controlling for error variance, it is also likely that these improved associations reflected the reorganization of the TriPM items to be conceptually and empirically aligned with the PCL scales. In contrast, the relationship between the 4FPM scales and the PCL-R in the incarcerated sample ranged from negligible to large, with the Interpersonal and Affective facets of the PCL-R displaying particularly low correlations with all domains of the 4FPM, concordant with Sleep et al. (2019) meta-analytic associations between these PCL-R domains and the original Triarchic scales (*r*’s = 0.10 to 0.27). It is possible that these attenuated correlations are due in part to differences in measurement (e.g., self-report vs. clinical rating) and a smaller incarcerated sample. Notably, using much larger samples, as well as latent variable modeling, Neumann et al. (2015) found stronger associations between self-reported and interviewer ratings of psychopathy. Still, in this latter study, the associations between Interpersonal and Affective features were smaller than those between self-reported and interviewer ratings of Lifestyle and Antisocial features. Thus, it is possible that incarcerated men in the current study and previous (Neumann et al., 2015) studies have limited insight into their interpersonal style and affective experiences. Research with a larger samples and positive impression management measures is warranted to clarify the associations between the 4FPM and the PCL-R.

The 4FPM and the SRP-SF scales displayed robust intercorrelations amongst themselves, which were modeled as a superordinate psychopathy factor for both measures. Model fit for the psychopathy superordinate model was acceptable and consistent with research on the PCL-R (Neumann et al., 2007). This indicates that total scores for both the SRP-SF and the 4FPM can be reliably calculated and utilized. Both superordinate factors accounted for a significant proportion of the variance in the respective four first-order factors, indicating that all four facets of psychopathy are essential to understanding the syndrome of psychopathy (Hare et al., 2018; Neumann et al., 2007). The psychopathy super factors for both measures evidenced near identical associations with external correlates in the community sample using structural equation modeling. These findings align with the profile similarity analyses in demonstrating that the four-factor model of psychopathic personality can be recovered from the TriPM items and function in a comparable manner to a PCL scale. Regarding relationships with external correlates, the psychopathy super factor was associated with increased alcohol use and negative affect, decreased positive affect, and negligible associations with trauma history. The current findings are consistent with previous research linking broad psychopathy propensities to substance use and emotional dysregulation (Garofalo et al., 2020; Neumann & Hare, 2008; Potik et al., 2019). The negligible associations with trauma history at the super factor level may be due in part to how these experiences were measured (i.e., self-reports of broad trauma history) in contrast to prior research indicating more fine-grained associations between specific forms of adverse experiences (i.e., physical, sexual abuse) and PCL-R facet scores (Graham et al., 2012). Finally, the current findings regarding positive affect mirror research linking the personality tendencies indexed by the SRP-SF with poor subjective well-being, including positive affect (Love & Holder, 2014).

The 4FPM demonstrated strong measurement invariance between the community and incarcerated samples and between genders in the community sample. These findings signify that the same underlying psychopathy domains are being tapped across community and incarcerated samples, even though in the latter, the likelihood of elevated psychopathic propensity is much greater. At the same time, evidence of strong (scalar) invariance was not supported when comparing community women with incarcerated men, a finding that is sensible given the differential prevalence rates of psychopathic traits between community and correctional samples as well as gender (Hare et al., 2018). Nonetheless, these findings are among the first to demonstrate that psychopathic propensities are being measured in a similar manner across community and correctional samples. However, further replication of these findings in larger and more diverse samples is warranted to ascertain the stability of the current findings.

Given good model fit and evidence of measurement invariance, sample means were examined to ascertain if differences in the expression of psychopathic propensities emerged as a function of whether the TriPM items were configured to align with the Triarchic Model of Psychopathy or the PCL-based four-factor structure. The original TriPM total score and scores on the Boldness and Meanness scales were higher in the community sample than in the incarcerated sample. This finding runs counter to the lower base rate of these traits in community relative to incarcerated settings (Hare & Neumann, 2008; Neumann & Hare, 2008). This highlights that the original TriPM scales may diminish understanding of the expression of psychopathic propensities in diverse settings as well as how these traits relate to external correlates (Roy et al., 2021a). Nonetheless, the items of the TriPM appear to have considerable value if reorganized into a more psychometrically sound structure, as evidenced by the ability of the 4FPM scales to better differentiate between incarcerated and community individuals.

Individuals within the incarcerated sample with elevated PCL-R scores did report significantly higher total TriPM scores, for both the original TriPM scale and the new 4FPM total scale, indicating some congruence between self-report and interviewer ratings of psychopathic features.^3^ Thus, if the goal is to identify persons with overall (syndromal) levels of psychopathic propensities, then total scores are useful, and the superordinate modeling results support the use of total scores (Neumann et al., 2007). However, if a primary goal of psychopathy research is to understand the correlates and etiological factors of the first-order trait domains, then total scores are non-optimal. Confirmatory and exploratory analyses of the TriPM item set across a variety of samples have revealed several items with poor psychometric properties (Roy et al., 2021b; Stanton et al., 2021). As such, employing a total score for the TriPM not only diverges from the assumptions of the Triarchic Model but also may have limited utility given the psychometric uncertainty inherent in the underlying subscales of the measure.

### 4.1. Implications

The current study has implications for the assessment of psychopathic tendencies using the TriPM. Specifically, it was demonstrated that a subset of items taken primarily from the Meanness and Disinhibition scales of the TriPM can be reconfigured to represent the PCL-R four-factor structure of psychopathic personality and function in a comparable manner to a well-validated self-report PCL scale. Given that such scales were developed from the Externalizing Spectrum Inventory, it is reasonable to expect that the four-factor proxy measure can be forged from this inventory as well, highlighting that psychopathic personality can be considered under the umbrella of the broader externalizing spectrum. However, this does not suggest that using the original Meanness and Disinhibition scales, which were shaped by taking the items that loaded onto latent factors indexing antagonistic and disinhibitory propensities in the ESI, respectively (Patrick et al., 2021), can measure psychopathic personality traits in the same manner as the PCL scales despite being composed of items relevant to psychopathy. This is consistent with Neumann et al.’s (2007) concern that integrating psychopathy with the broader externalizing constructs may lead to a dilution of the initial conceptualization. In the case of the TriPM, the poor discriminant validity of the original Meanness and Disinhibition may reflect such an occurrence (Roy et al., 2021b; Sleep et al., 2019).

As the four-factor proxy measure was created from the TriPM, it is possible that this measure may be derived from the items of the Triarchic derivative scales forged from other psychopathy and multi-scale inventories, such as the Minnesota Multiphasic Personality Inventory-2 Restructured Form-Triarchic scales (MMPI-2RF-Tri: Kutchen et al., 2016). Although further validation of this measure and future four-factor proxy measures is warranted, such scales would allow researchers and clinicians to assess the PCL-R model of psychopathy in tandem with other constructs of relevance to clinical and forensic decision-making and compare such score profiles with available PCL-R ratings or other treatment or forensically relevant instruments.

Although the 4FPM derived in the current study functioned in a manner comparable to the SRP-SF, it is not recommended that this measure replace either the TriPM or the SRP-SF. The reliability and validity of the 4FPM are currently constrained to the parameters of this study (e.g., sample demographics, variables examined). As such, the 4FPM requires further validation to assess its convergence and divergence with other psychopathy measures. Additionally, we do not advocate for the 4FPM replacing the use of the TriPM, given that the TriPM is intended to assess constructs through the lens of the Triarchic Model of Psychopathy, which the PCL scales do not. Nonetheless, the 4FPM can also be utilized to complement Triarchic research aims. For example, as Boldness items are not represented extensively in the 4FPM, researchers can examine if the Boldness scale, or the three unidimensional scales from the seven-factor model, can provide incremental validity to the prediction of external correlates using the 4FPM.

### 4.2. Limitations and Future Directions

The current study has several notable limitations. Specifically, the correctional sample was relatively small for latent variable modeling, contained only European men, and did not have any external correlate data beyond the PCL-R. Similarly, the external correlate data available in the community sample were limited and circumscribed to self-report data. Future research would benefit from deriving the 4FPM in large TriPM samples with diverse sample demographics, psychopathy measures, personality inventories, and external correlates (e.g., neurocognitive variables, psychophysiological measures, criminal justice variables). Additionally, the current study utilized confirmatory factor analysis, which some researchers have criticized as having unrealistic assumptions for use in personality research (e.g., Sellbom & Tellegen, 2019). As such, future research utilizing exploratory structural equation modeling may be helpful to continue examining the psychometric properties of the 4FPM. Furthermore, item selection for the 4FPM was informed by psychometric examinations of the TriPM in two datasets and one of the author’s considerable expertise in modeling the PCL scales. As such, future research may benefit from utilizing the item selection techniques (i.e., consensus rating) employed in the Triarchic proxy scale literature (e.g., Kutchen et al., 2016) to assess the extent to which other raters agree that the 4FPM item set sufficiently indexes the traits and tendencies captured by the PCL scales.

In summary, the current study highlighted that selected items of the TriPM can be reconfigured into a measure that reliably indexed the latent structure of PCL-R psychopathic personality, assessed the construct in a generally similar manner across sample settings and gender demographics, and functioned in a manner analogous to the SRP-SF. Although this proxy measure requires further validation, the derivation of a psychopathy measure with a clear, psychometrically sound internal structure can provide a complementary measure to researchers using the TriPM item set, as that measure continues to be refined to advance our understanding of a construct of paramount importance to mental health and criminal justice systems.

## Figures and Tables

**Table 1:** Profile similarity analyses between the SRP-SF scales and the parallel 4FPM scales in the community sample.

Psychopathy Factor	Shape Similarity	Elevation (Dis)Similarity	Scatter (Dis)Similarity	*rICC*
Interpersonal Scales	0.96	0.01	0.001	0.95
Affective Scales	0.95	0.05	0.002	0.94
Lifestyle Scales	0.92	0.03	0.002	0.91
Antisocial Scales	0.94	0.01	0.004	0.94

Note: *rICC* = double-entry intraclass correlation.

**Table 2:** SEM results for 4FPM and SRP-SF superordinate models.

4FPM Superordinate Model with Latent Regressions		
**Scale loading on the superordinate factor**	β	*R* ^2^
*4FPM-Interpersonal*	**0.93**	**0.87**
*4FPM-Affective*	**0.85**	**0.72**
*4FPM-Lifestyle*	**0.86**	**0.74**
*4FPM-Antisocial*	**0.91**	**0.83**
**Criterion**		
*Alcohol Use*	**0.39**	**0.16**
Trauma History Questionnaire	0.04	0.00
PANAS-Positive	**−0.18**	*0.03*
PANAS-Negative	**0.31**	**0.09**
**SRP-SF Superordinate Model with Latent Regressions**		
**Scale loading on the superordinate factor**	β	*R* ^2^
*SRP-Interpersonal*	**0.94**	**0.88**
*SRP-Affective*	**0.94**	**0.88**
*SRP-Lifestyle*	**0.88**	**0.78**
*SRP-Antisocial*	**0.88**	**0.77**
**Criterion**		
*Alcohol Use*	**0.39**	**0.15**
Trauma History Questionnaire	*0.09*	0.01
PANAS-Positive	**−0.15**	0.02
PANAS-Negative	**0.27**	**0.07**

Note: Italicized variable names indicate a latent variable. SRP = Self-Report Psychopathy Scale; PANAS = Positive and Negative Affective Schedule. Underlined = *p* < 0.05; underline italics = *p* < 0.01; bold = *p* < 0.001.

**Table 3:** Multiple group confirmatory factor analysis (MG-CFA): measurement invariance results.

		Configural			Strong (Scalar) Invariance		
Model Fit	CFI	RMSEA	SRMR	CFI	RMSEA	SRMR	ΔCFI
Groups							
		**TriPM items four-factor (4FPM) model**					
Com. Males/Females	0.94	0.06	0.07	0.94	0.05	0.07	0.00
Com./Incarcerated	0.94	0.07	0.06	0.94	0.06	0.06	0.00
Male Com./Incarcerated	0.94	0.05	0.06	0.94	0.05	0.06	0.00
Female Com./Incarcerated	0.93	0.06	0.08	0.91	0.06	0.08	0.02
		**SRP-SF four-factor model**					
Com. Males/Females	0.91	0.07	0.08	0.91	0.07	0.08	0.00

Note: All invariance tests provided evidence for strong invariance, except between men convicted of criminal offenses and community females, which fell slightly outside of the recommended CFI difference of ≥0.01.

**Table 4:** Multiple group confirmatory factor analysis: standardized factor loadings four-factor PCL-based proxy model using select TriPM item sets.

	Community	Incarcerated	Com. Male	Com. Female
Interpersonal				
23M: Enjoy pushing people…	0.80	0.75	0.80	0.73
26M: Taunt people to stir up…	0.89	0.80	0.87	0.89
34D: Conned to get money…	0.83	0.67	0.82	0.86
38B: Convince people to do…	0.31	0.61	0.25	0.32
42M: Insult people for fun…	0.84	0.83	0.83	0.83
Affective				
14M: Enjoy physical fight…	0.71	0.78	0.61	0.80
29M: Not worry if I hurt people…	0.80	0.95	0.79	0.77
36M: No sympathy for people…	0.70	0.74	0.67	0.68
48M: Injure people to see pain…	0.87	0.97	0.87	0.84
40M: Don’t care if I hurt people…	0.86	0.75	0.86	0.90
55M: Not bothered if people hurt…	0.76	0.84	0.73	0.76
Lifestyle				
3D: Act on immediate needs…	0.36	0.33	0.36	0.40
9D: Impulsive decisions…	0.82	0.65	0.80	0.85
15D: Do without thinking…	0.73	0.69	0.74	0.71
31D: Get bored quickly…	0.55	0.59	0.58	0.53
37D: Not consider consequences…	0.80	0.66	0.82	0.79
45M: Things fun if dangerous…	0.64	0.73	0.53	0.70
51D: Lack self-control…	0.86	0.73	0.87	0.82
Antisocial				
24D: Take people’s money…	0.78	0.76	0.81	0.72
43D: Steal from stores…	0.64	0.81	0.66	0.63
53D: Robbed people…	0.87	0.84	0.83	0.96
56D: Irresponsible at work…	0.71	0.74	0.66	0.71
58D: Stole things from car…	0.85	0.92	0.81	0.91

Note: B = Boldness scale item; M = Meanness scale item; D = Disinhibition scale item. All parameters are *p* < 0.001.

**Table 5:** Mean item scores, standard deviations (SDs), and significant differences by sample type (incarcerated vs. community).

**Scales**	**Sample Type**					
	**Incarcerated (I)**		**Community (C)**			
**Original TriPM**	**Mean**	**SD**	**Mean**	**SD**	**I vs. C**	
Boldness	1.46	0.46	1.59	0.23	F(1,1207) = 33.40, *p* < 0.001, *n*^2^ = 0.03	
Meanness	0.73	0.57	1.02	0.31	F(1,1207) = 93.69, *p* < 0.001, *n*^2^ = 0.07	
Disinhibition	1.33	0.65	0.91	0.43	F(1,1208) = 106.67, *p* < 0.001, *n*^2^ = 0.08	
**4FPM**						
Interpersonal	0.61	0.61	0.60	0.56	F(1,1208) = 0.13, *ns*, *n*^2^ = 0.00	
Affective	0.60	0.70	0.39	0.50	F(1,1207) = 20.15, *p* < 0.001, *n*^2^ = 0.02	
Lifestyle	1.53	0.74	0.78	0.64	F(1,1207) = 176.95, *p* < 0.001, *n*^2^ = 0.13	
Antisocial	1.34	1.04	0.40	0.56	F(1,1207) = 287.46, *p* < 0.001, *n*^2^ = 0.18	
**Scales**	**Sample Type**					
	**Com. Men** **(CM)**		**Com. Women** **(CW)**			
**Original TriPM**	**Mean**	**SD**	**Mean**	**SD**	**I vs. CM**	I vs. CW *
Boldness	1.61	0.23	1.57	0.23	F(1,705) = 33.26, *p* < 0.001, *n*^2^ = 0.04	F(1,650) = 16.89, *p* < 0.001, *n*^2^ = 0.02
Meanness	1.09	0.34	0.95	0.25	F(1,705) = 99.30, *p* < 0.001, *n*^2^ = 0.12	F(1,650) = 45.45, *p* < 0.001, *n*^2^ = 0.06
Disinhibition	0.96	0.45	0.86	0.39	F(1,705) = 63.20, *p* < 0.001, *n*^2^ = 0.08	F(1,650) = 118.95, *p* < 0.001, *n*^2^ = 0.15
**4FPM**						
Interpersonal	0.72	0.59	0.46	0.48	F(1,705) = 3.99, *p* < 0.05, *n*^2^ = 0.00	F(1,650) = 11.17, *p* < 0.001, *n*^2^ = 0.02
Affective	0.52	0.54	0.24	0.40	F(1,705) = 2.08, *ns*, *n*^2^ = 0.00	F(1,650) = 60.52, *p* < 0.001, *n*^2^ = 0.08
Lifestyle	0.88	0.64	0.66	0.61	F(1,705) = 112.38, *p* < 0.001, *n*^2^ = 0.14	F(1,650) = 213.43, *p* < 0.001, *n*^2^ = 0.25
Antisocial	0.49	0.60	0.30	0.49	F(1,705) = 168.00, *p* < 0.001, *n*^2^ = 0.19	F(1,650) = 125.85, *p* < 0.001, *n*^2^ = 0.31

Note: * Incarcerated men vs. community women comparison did not achieve strict invariance; thus, mean comparisons should be interpreted with caution. Underlined means for unexpected differences between incarcerated and community samples. Community men and women differed in the expected direct (M > W), except that there was a difference in Boldness scores.

## Data Availability

The raw data supporting the conclusions of this article will be made available by the authors on request.

## Notes

1 Briefly, measurement invariance is conducted in a stepwise fashion to evaluate which comparisons across groups can be made (Maassen et al., 2023). Configural invariance testing indicates the extent to which the number of latent variables and factor loading patterns are the same in all groups. Metric or weak invariance establishes whether factor loadings are measured equivalently across groups and can be compared. Scalar or strong invariance testing examines whether item intercepts or thresholds are invariant, allowing for latent mean comparisons between groups (Maassen et al., 2023).

2 Per Furr (2010), a profile can be characterized by three fundamental elements: elevation (i.e., the average score across all variables in the profile), scatter (i.e., the variability among the scores in a profile), and shape (i.e., the pattern of scores in a profile). According to Furr (2010), the intra-class correlation can be viewed as an omnibus test of profile similarity as it taps into all three of the aforementioned elements. In light of this multidimensional indicator, it is recommended each component be examined separately when evaluating profile similarity.

3 It is worth noting that we also examined how individuals with elevated PCL-R scores differed from those with non-elevated PCL-R scores, and found that there was no difference in Boldness scores, though those with elevated PCL-R scores did report higher Meanness and Disinhibition scores, as well as higher 4FPM Affective, Lifestyle, and Antisocial scores.

## Supplementary Materials

The following supporting information can be downloaded at https://www.mdpi.com/article/10.3390/bs15111503/s1, Table S1: Latent and manifest correlations among variables in the community sample; Table S2: Latent and manifest psychopathy scale correlations for the incarcerated men sample.
