# Supplementary material for: Validating a Four-Factor Model of Psychopathic Personality from the Triarchic Psychopathy Measure (TriPM) Across Community and Incarcerated Samples
Source: Behav Sci (Basel). 2025 Nov 5;15(11):1503. doi: 10.3390/bs15111503 (PMC12649613; doi:10.3390/bs15111503)
Supplement: Supplementary file 1 [file behavsci-15-01503-s001.zip › behavsci-3749225-supplementary.pdf]

Supplemental Tables

Supplementary Table S1.
Latent and manifest correlations among variables in community sample.

Variables	1.	2.	3.	4.	5.	6.	7.	8.	9.	10.	11.	12.
1. SRP-Interpersonal												
2. SRP-Affective	*.90*											
3. SRP-Lifestyle	*.80*	*.84*										
4. SRP-Antisocial	*.84*	*.78*	*.80*									
5. 4FPM-Interpersonal	***.82***	*.76*	*.71*	*.72*								
6. 4FPM-Affective	*.70*	***.87***	*.61*	*.68*	*.87*							
7. 4FPM-Lifestyle	*.64*	*.67*	***.85***	*.60*	*.76*	*.70*						
8. 4FPM-Antisocial	*.71*	*.63*	*.71*	***.82***	*.82*	*.73*	*.83*					
9. Alcohol Use (AUDIT)	*.33*	*.32*	*.41*	*.34*	*.32*	*.25*	*.40*	*.42*				
10. PANAS-Negative	*.12*	*.24*	*.24*	*.20*	*.21*	*.17*	*.37*	*.31*	*.12*			
11. PANAS-Positive	*-.04*	*-.23*	*-.06*	*-.09*	*-.04*	*-.18*	*-.21*	*-.19*	*-.04*	-.25		
12. Trauma History Questionnaire	*.07*	*.09*	*.13*	*.06*	*.02*	*-.07*	*.13*	*.10*	*.07*	.24	.01	

Note. *Italics* = Latent correlations. **Bold** = Convergent Validity Associations. 4FPM = Four Factor Proxy Measure; SRP = Self-Report Psychopathy factor(s). Correlations of |.06| are statistically significant at $p < .05$ level, correlations of |.10| are statistically significant at $p < .01$ level, correlations of |.12| are statistically significant at $p < .001$

Supplementary Table S2.
Latent and manifest psychopathy scale correlations for incarcerated men sample.

Variables	1	2	3	4	5	6	7	8	9
1. 4FPM-Interpersonal									
2. 4FPM-Affective	*.82*								
3. 4FPM-Lifestyle	*.69*	*.78*							
4. 4FM-Antisocial	*.68*	*.53*	*.67*						
5. PCL-R Interpersonal	**.01**	.06	-.03						
6. PCL-R Affective	.05	**.13**	.06		.59				
7. PCL-R Lifestyle	.27	.27	**.42**		.30	.39			
8. PCL-R Antisocial	.29	.18	.40	**.50**	.33	.37	.62		
9. PCL-R Total	.22	.21	.29	.37	.68	.77	.76	.79	

Note. *Italics* = Latent correlations. **Bold** = Convergent Validity Associations. 4FPM = Four Factor Proxy Measure; PCL-R = Psychopathy Checklist-Revised. Correlations of |.18| are statistically significant at $p < .05$ level, correlations of |.21| are statistically significant at $p < .01$ level, correlations of |.29| are statistically significant at $p < .001$.
